# Supplementary material for: Direct catalytic hydrogenation of CO2 to formate over a Schiff-base-mediated gold nanocatalyst
Source: Nat Commun. 2017 Nov 10;8:1407. doi: 10.1038/s41467-017-01673-3 (PMC5680246; doi:10.1038/s41467-017-01673-3)
Supplement: Supplementary file 1 — Supplementary information [file 41467_2017_1673_MOESM1_ESM.pdf]

Supplementary Methods

Preparation of unfunctionalized Au/SiO_2 Catalyst

Firstly, Au/SiO_2-NH_2 with a theory loading of 1.5 wt.% was prepared by the same synthetic procedure as described in the manuscript. Subsequently, the obtained gold catalyst (0.3 g) was redispersed in 400 mL ethanol solutions, to which 50 μL of TEOS and 1.0 mL of ammonium hydroxide solution (28 wt%) were added under continuous stirring. After stirring at room temperature for 6 h, the precipitate was filtered and washed with deionized water. Following drying in vacuum at 120 ^{o}C for 12 h, Au/SiO_2-NH_2 catalyst was obtained. The achieved catalysts were then calcined at 800 ^{o}C for 20 s at O_2 atmosphere to obtain the unfunctionalized Au/SiO_2 catalyst.

Calculations of turnover number (TON) and turnover frequency (TOF)

After ensuring that the hydrogenation is free of diffusion limitations (e.g. varying stirrer speed and catalyst mass, the selected stirrer speed is as high as 600 r/min and the catalyst was grinded before use), the TON and initial TOF reported here is calculated by the equation as follows:

$$TON = \frac{C_{HCOOH} \times V}{n_{Au}} \quad (1)$$

$$TOF = \frac{C_{HCOOH} \times V}{n_{Au}\, t} \quad (2)$$

Where C_{HCOOH} is the formate concentration monitored by HPLC, n_{Au} is the total mole number of Au atoms in catalyst, *V* is the volume of reactants, and *t* is the initial time of the catalytic reaction (where C_{HCOOH} is less than 0.05 M compared with the 1.5 M base added).

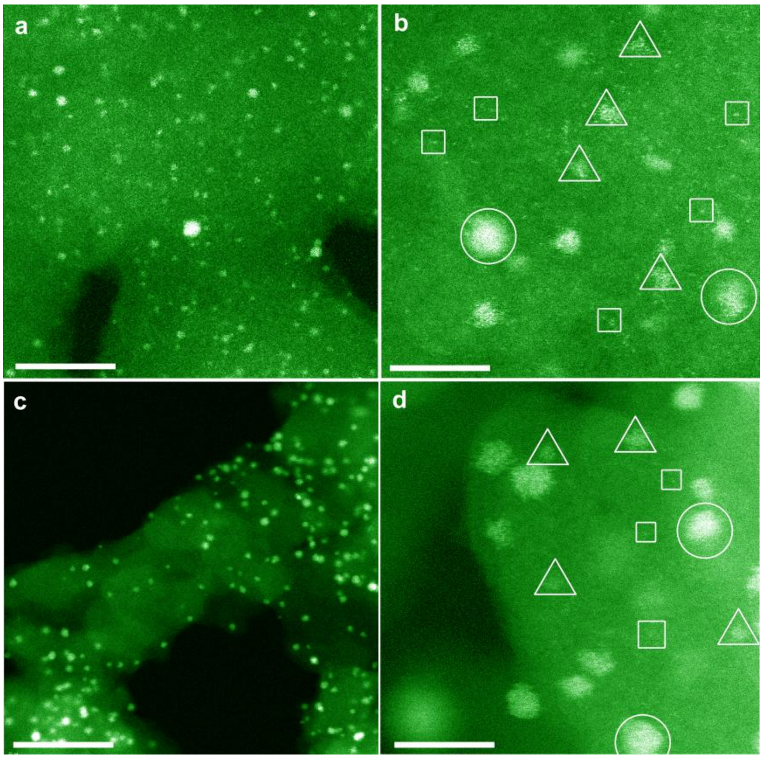

Supplementary Figure 1. (a) Typical aberration-corrected HAADF-STEM images of Au/SiO_2-Schiff (a,b) and Au/SiO_2-NH_2 (c,d). Scale bars in **a,c** represent 20 nm. Scale bars in **b,d** represent 5 nm. The Au species are mainly dispersed as nanoclusters (triangle), as well as single atoms (square) and nanoparticles larger than 2 nm (circles).

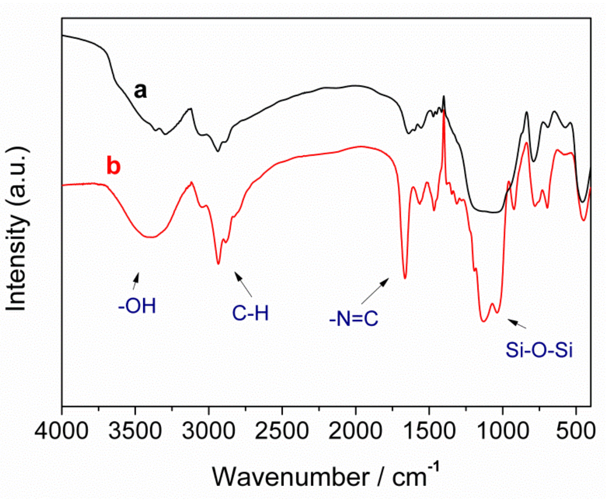

Supplementary Figure 2. FTIR spectra of the functionalized gold catalysts. (**a**) Au/SiO_2-NH_2 catalyst. (**b**) Au/SiO_2-Schiff catalyst.

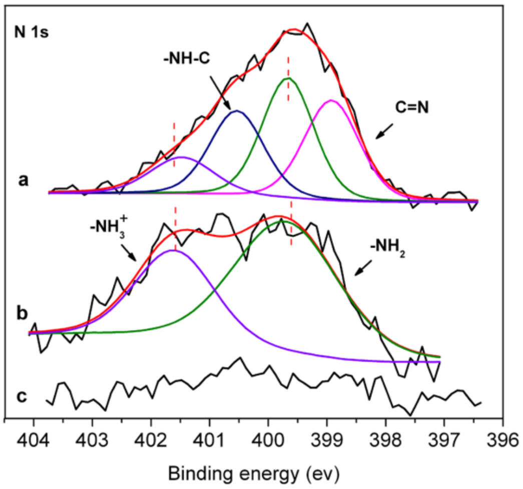

Supplementary Figure 3. XPS spectra of the N 1s core levels of the as-prepared gold catalysts. (**a**) Au/SiO_2-Schiff, (**b**) Au/SiO_2-NH_2 and (**c**) unfunctionalized Au/SiO_2.

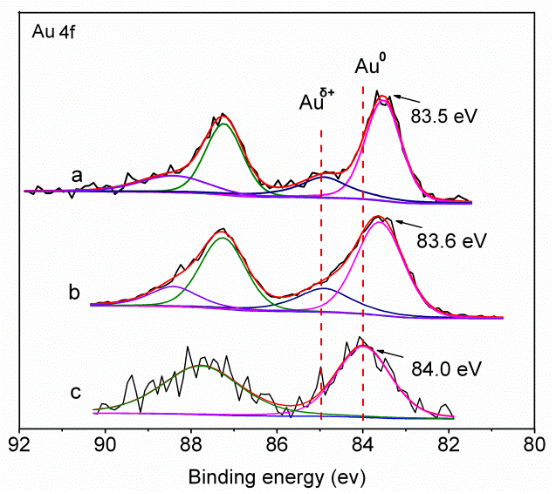

Supplementary Figure 4. XPS spectra of the Au 4f core levels of the as-prepared gold catalysts. (**a**) Au/SiO_2-NH_2, (**b**) Au/SiO_2-Schiff and, (**c**) unfunctionalized Au/SiO_2. Besides metallic gold, both the functionalized samples contained some form of cationic Au species that were ascribed to sub-nanometric cationic Au clusters and isolated Au atoms.

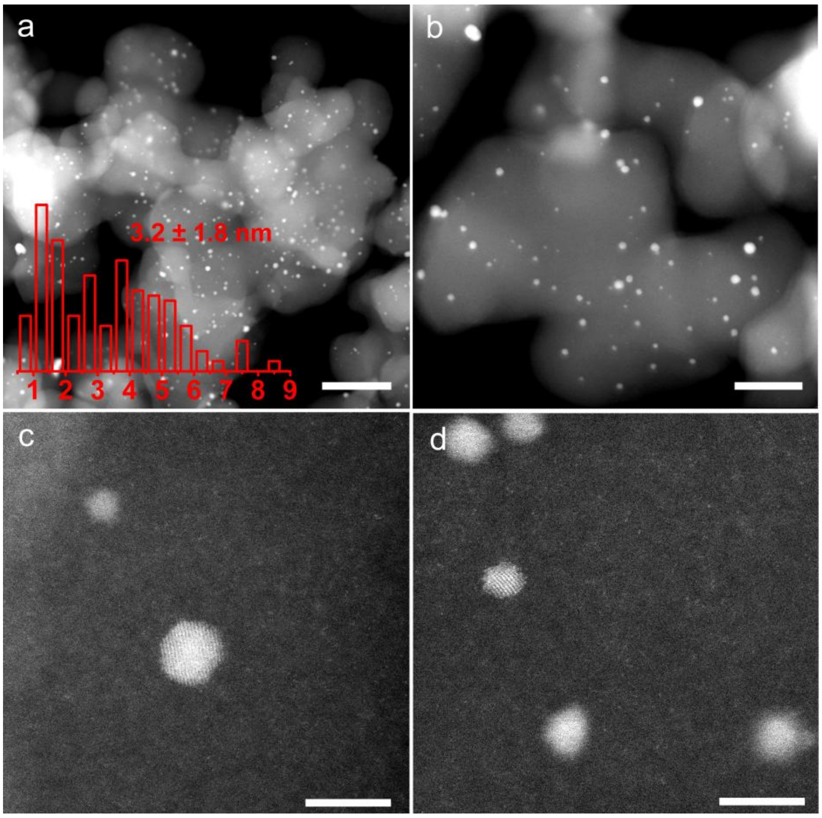

Supplementary Figure 5. Typical HAADF-STEM images of Au/SiO_2-Schiff catalyst prepared by *in situ* reduction method. Scale bars in **a** represent 100 nm. Scale bars in **b** represent 50 nm. Scale bars in **c,d** represent 5 nm. The Au species are mainly dispersed as nanoparticles larger than 2 nm and some individual atoms.

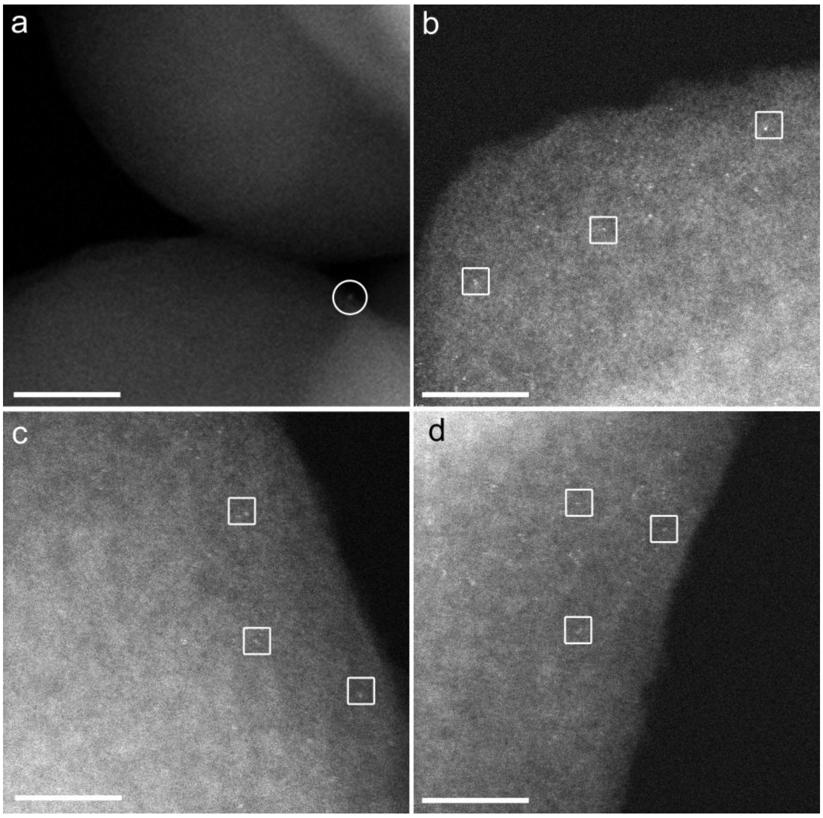

Supplementary Figure 6. Typical HAADF-STEM images of Au/SiO_2-Schiff catalyst prepared by the solid reduction method. Scale bars in **a** represent 20 nm. Scale bars in **b, c** and **d** represent 5 nm. The figures showed that the Au species are mainly dispersed as single atoms (white squares), as well as some negligible nanoclusters (white circle).

Adsorption patterns	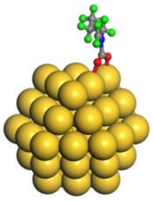	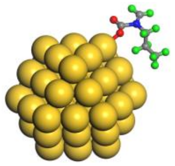	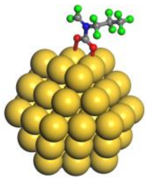	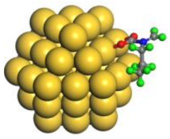	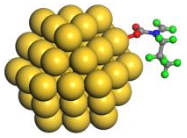
**E_{ad} (eV)**	**-0.40**	**-0.38**	**-0.23**	**-0.29**	**-0.24**
**$\nu_{C\text{-}O}$ (cm^{-1})**	**1701**	**1767**	**1752**	**1741**	**1757**

Supplementary Figure 7. Theoretical understanding the chemisorption of CO_2 on the model of Au/SiO_2-Schiff catalyst with an Au_{55} cluster. The simulation was performed with the program package $DMol^3$ in the Materials Studio of Accelrys Inc. Geometry with a closed packed Au_{55} nanocluster (ca ~1.2 nm) and a silylation imine Schiff-base in COSMO of water was used to model the practical Au/SiO_2-Schiff catalyst in water.

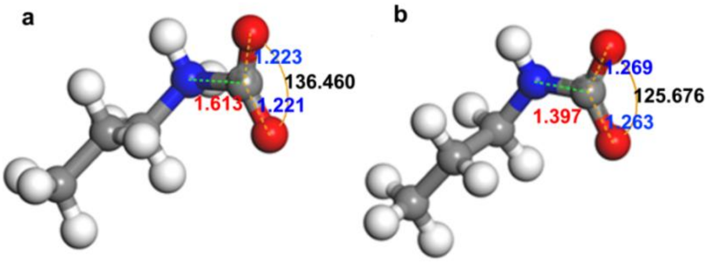

Supplementary Figure 8. Activation patterns of CO_2 through carbamate zwitterion (a) or ionic carbamate (b) species. The chemical interaction between CO_2 and propylamine is modelling with NWchem software at a B3LYP/def2-tzvp level.

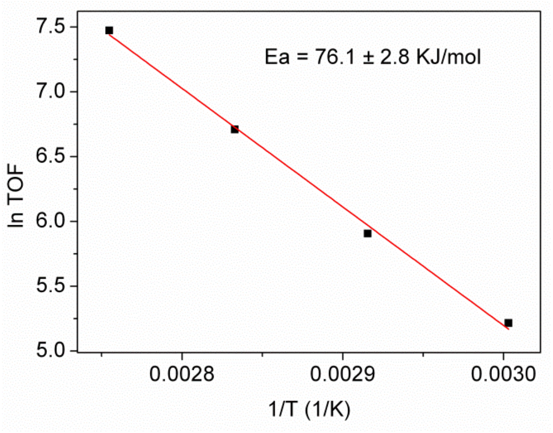

Supplementary Figure 9. Arrhenius plot for Au/SiO_2-Schiff catalyzed hydrogenation of CO_2 (lnTOF vs. 1/T). Reaction conditions: 5.0 mg catalyst, 10 mL H_2O-MeOH (20/80 v/v), 10 mmol NEt_3, $P(H_2)/P(CO_2)$ = 5.0/3.0 MPa, 600 rpm.

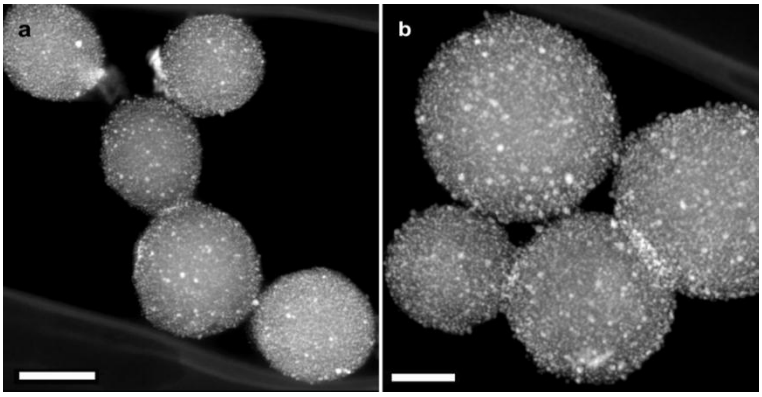

Supplementary Figure 10. HAADF-STEM images of Au/SiO_2-Schiff after catalytic hydrogenation of CO_2. Scale bars in **a** represent 100 nm. Scale bars in **b** represent 50 nm.

Supplementary Table 1. Metal loadings and surface atomic ratios of the prepared catalysts.

Catalyst	Metal loading [a] (wt.%)	atomic ratios (%) [b]		
		C	Au	N
Au/SiO_2-NH_2	1.62	22.66	0.39	3.38
Au/SiO_2-Schiff	1.41	51.57	0.16	9.16
Au/SiO_2-Schiff [c]	0.7	52.77	0.10	7.51
Au/SiO_2	1.50	21.95	0.15	1.08

[a] The actual metal loading was measured by inductively coupled plasma mass spectrometry (ICP-AES).

[b] The atomic ratios were measured by XPS.

[c] The catalyst was prepared by *in situ* reduction method.

Supplementary Table 2. Catalytic activity of the Au/SiO_2-Schiff catalyst for CO_2 hydrogenation in water/methanol mixtures.[a]

Entry	H_2O/CH_3OH v/v (%)	$P(H_2/CO_2)$ MPa	Base	Time /h	HCOOH /M	TON
1	0	5.0/3.0	NEt_3	12	0.702	9806
2	5	5.0/3.0	NEt_3	12	0.801	11189
3	10	5.0/3.0	NEt_3	12	0.814	11370
4	20	5.0/3.0	NEt_3	12	0.908	12684
5	40	5.0/3.0	NEt_3	12	0.819	11440
6[b]	20	5.0/3.0	NEt_3	12	0.518	14470

[a] Reaction conditions: 10 mg catalyst, 90 ^{o}C, 10 mL reagent, 15 mmol base, 600 rpm. [b] 5 mg Au/SiO_2-Schiff.

Supplementary Table 3. Catalytic activities for hydrogenation of CO_2 to formic acid catalyzed by recently reported heterogeneous and homogeneous catalysts.[a]

Catalyst	Temp. /^oC	Base	p(H_2/CO_2) (MPa)	Time /h	TON	TOF [c] /h^{-1}	Ref.
**Heterogeneous catalysts**							
**Au/SiO_2-Schiff**	**90**	**NEt_3**	**5/3**	**12**	**14,470**	**1,206**	**This work**
AURO*lite* [b]	40	NEt_3	9/9	37 days	18,040	28	1
Au NP/Al_2O_3	70	NEt_3	2/2	20	215	10	2
Au NP/TiO_2	70	NEt_3	2/2	20	111	5.5	2
Au NP/CeO_2	70	NEt_3	2/2	20	8	<1	2
Pd/C_3N_4	150	NEt_3	2.7/1.3	24	106	4.4	3
bpy-CTF-[IrCp*Cl]Cl	120	NEt_3	4/4.	2	5,000	2,500	4
IrPN/SBA-15	60	NEt_3	2/2	20	2,700	135	5
Ir(III)/COFs	120	NEt_3	4/4	10	6,400	640	6
Pd/r-GO	100	$KHCO_3$	4/0	32	7,088	221	7
Pd/C_3N_4	60	$KHCO_3$	6/0	5	6,595	1,319	8
Ru/LDH (Mg^{2+}/Al^{3+}=5)	100	NaOH	1/1	24	698	29	9
**Homogeneous catalysts**							
$RhCl(PPh_3)_3$	25	NEt_3	2/4	20	2,500	125	10
$RuCl(OAc)(PMe_3)_4$	50	NEt_3/ C_6F_5OH	7/12	0.33	32,000	95,000	11
$[RuCl_2(tppms)_2]_2$	50	$NaHCO_3$	1/0	6	180	50	12
$[RuCl_2(C_6H_6)]$/dppm	70	$NaHCO_3$	5/3.5	2	2,520	1,260	13
IrH_3(P1)	120	KOH	4/4	48	3500,000	73,000	14
IrH_3(P2)	185	KOH	2.8/2.8	24	348,000	14,500	15
Ru(P3)CO(H)	200	K_2CO_3	3/1	48	23,000	2,200	16
$[Rh(cod)(methallyl)_2]$/ PBu_4tppms	50	NEt_3	5/5	20	310	630	17
$[RuCl_2(P(OMe)_3)_4]$	100	DBU/ C_6F_5OH	7/10	4	6,630	1,660	18
$[Cp^*Ir(4DHBP)Cl]^+$	120	KOH	3/3	57	190,000	(42,000)	19
$[Cp^*Ir(6DHBP)(OH_2)]^{2+}$	120	$KHCO_3$	0.5/0.5	8	12,500	(25,200)	20
$[(Cp^*IrCl)_2(THBPM)]^{2+}$	50	$KHCO_3$	2/2	8	153,000	(15,700)	21
$[Cp^*Ir(N1)(OH_2)]^{2+}$	80	$KHCO_3$	1.5/1.5	8	34,000	(33,300)	22
IrI_2(AcO)(bis-NHC)	200	KOH	3/3	75	190,000	2,500	23
Fe(PNP^{Me}-*i*Pr)(H)(CO)(Br)	80	DBU	0.4/0.4	21	10,275	489	24

[a] Abbreviations are the following: PC= bidentate iminophosphine ligand, dppm = 1,1-bis(diphenylphosphino)methane, cod = 1,5-cyclooctadiene, P1 = 2,6-bis(diisopropylphosphinomethyl)pyridine,

P2 = (diisopropylphoshinoethyl)amine, P3 = 6-(di-tertbutylphosphinomethylene)-2-(N,N-diethylaminomethyl)-1,6-dihydropyridine, N1 = 2,2′,6,6′-tetrahydroxy-4,4′-bipyrimidine. PNP = 2,6-bis(di-tert-butylphosphinomethyl)pyridine. DBU= 1,8-Diazabicyclo[5.4.0]undec-7-ene. [b] commercial AUROlite (Mintek; 1 wt% Au/TiO_2, extrudates). [c] The data in the parentheses are initial TOFs.

Supplementary Table 4. Catalytic hydrogenation of CO_2 to formic acid by Au/SiO_2-Schiff at different temperatures (60–90 ^oC).

Entry	Temp. /^{o}C	Time /min	HCOOH/M	TOF
1	60	120	0.014	195
2	70	90	0.019	353
3	80	50	0.028	938
4	90	30	0.035	1950

Reaction conditions: 5.0 mg catalyst, 10 mL H_2O-MeOH (20/80 v/v), 10 mmol NEt_3, $P(H_2)/P(CO_2)$ = 5.0/3.0 MPa, 600 rpm.

Supplementary Table 5. Hydrogenation of gaseous CO_2 by Au/SiO_2-Schiff.

Entry	Base	$P(H_2)/P(CO_2)$ MPa	HCOOH /M	TON
1	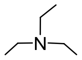	6.0/2.0	0.29	8102
2	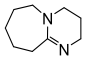	6.0/2.0	0.23	6425
3	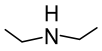	6.0/2.0	0.15	4190
4	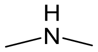	6.0/2.0	0.067	1871
5	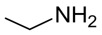	6.0/2.0	0.08	2235

Reaction conditions: 5.0 mg catalyst, 90 ^{o}C, 10 mL H_2O-MeOH (20/80 v/v), 10 mmol base, 6 h, 600 rpm.

Supplementary Table 6. Reuse of the Au/ SiO_2-Schiff catalyst for the hydrogenation of CO_2 to formic acid.

Cycle	HCOOH /M	TON
1	0.908	12684
2	0.652	9107
3	0.424	5922

Reaction conditions: 10 mg catalyst, 90 ^{o}C, 10 mL reagent, 15 mmol base, 12 h, 600 rpm.

Supplementary References:

[1] Preti, D., Resta, C., Squarcialupi, S. & Fachinetti, G. Carbon dioxide hydrogenation to formic acid by using a heterogeneous gold catalyst. *Angew. Chem. Int. Ed*. **50,** 12551−12554 (2011).

[2] Filonenko, G. A., Vrijburg, W. L., Hensen, E. J. & Pidko, E. A. On the activity of supported Au catalysts in the liquid phase hydrogenation of CO_2 to formates. *J. Catal*. **343**, 97–105 (2016).

[3] Lee, J. H. *et al.* Carbon dioxide mediated, reversible chemical hydrogen storage using a Pd nanocatalyst supported on mesoporous graphitic carbon nitride. *J. Mater. Chem. A* **2,** 9490–9495 (2014).

[4] Park, K., Gunasekar, G. H., Prakash, N., Jung, K. D. & Yoon, S. A highly efficient heterogenized iridium complex for the catalytic hydrogenation of carbon dioxide to formate. *ChemSusChem* **8,** 3410−3413 (2015).

[5] Xu, Z., McNamara, N. D., Neumann, G. T., Schneider, W. F. & Hicks, J. C. Catalytic hydrogenation of CO_2 to formic acid with silica-tethered iridium catalysts. *ChemCatChem* **5,** 1769−1771 (2013).

[6] Hariyanandam, G. G., Hyun, D., Natarajan, P., Jung, K. D. & Yoon, S. An effective heterogeneous Ir (III) catalyst, immobilized on a heptazine-based organic framework, for the hydrogenation of CO_2 to formate. *Catal. Today* **265,** 52−55 (2016).

[7] Bi, Q. Y., Lin, J. D., Liu, Y. M., Du, X. L., Wang, J. Q., He, H. Y. & Cao, Y. An aqueous rechargeable formate–based hydrogen battery driven by heterogeneous Pd catalysis. *Angew. Chem. Int. Ed.* **53,** 13583−13587 (2014).

[8] Shao, X., Xu, J., Huang, Y., Su, X., Duan, H., Wang, X. & Zhang, T. Pd@ C_3N_4 nanocatalyst for highly efficient hydrogen storage system based on potassium bicarbonate/formate. *AIChE J.* **62,** 2410-2418 (2016).

[9] Mori, K., Taga, T. & Yamashita, H. Isolated single-atomic Ru catalyst bound on a layered double hydroxide for hydrogenation of CO_2 to formic acid. *ACS Catal.* **7,** 3147−3151 (2017).

[10] Ezhova, N. N., Kolesnichenko, N. V., Bulygin, A. V., Slivinskii, E. V. & Han, S. Hydrogenation of CO_2 to formic acid in the presence of the Wilkinson complex. Russ. *Chem. Bull.* **51,** 2165−2169 (2002).

[11] Munshi, P., Main, A. D., Linehan, J. C., Tai, C. C. & Jessop, P. G. Hydrogenation of carbon dioxide catalyzed by ruthenium trimethylphosphine complexes: The accelerating effect of certain alcohols and amines. *J. Am. Chem. Soc.* **124,** 7963−7971 (2002).

[12] Elek, J., Nádasdi, L., Papp, G., Laurenczy, G. & Joó, F. Homogeneous hydrogenation of carbon dioxide and bicarbonate in aqueous solution catalyzed by water-soluble ruthenium(II) phosphine complexes. *Appl. Catal. A Gen.* **255,** 59−67 (2003).

[13] Federsel, C., Jackstell, R., Boddien, A., Laurenczy, G. & Beller, M. Ruthenium-catalyzed hydrogenation of bicarbonate in water. *ChemSusChem* **3,** 1048−1050 (2010).

[14] Tanaka, R., Yamashita, M. & Nozaki, K. Catalytic hydrogenation of carbon dioxide using Ir(III)-pincer complexes. *J. Am. Chem. Soc*. **131,** 14168−14169 (2009).

[15] Schmeier, T. J., Dobereiner, G. E., Crabtree, R. H. & Hazari, N. Secondary coordination sphere interactions facilitate the insertion step in an Iridium(III) CO_2 reduction catalyst. *J. Am. Chem. Soc.*, **133,** 9274−9277 (2011).

[16] Huff, C. A. & Sanford, M. S. Catalytic CO_2 hydrogenation to formate by a ruthenium pincer complex. *ACS Catal.* **3,** 2412−2416 (2013).

[17] Wesselbaum, S., Hintermair, U. & Leitner, W. Continuous-flow hydrogenation of carbon dioxide to pure formic acid using an integrated $scCO_2$ process with immobilized catalyst and base. *Angew. Chem., Int. Ed.* **51,** 8585−8588 (2012).

[18] Muller, K., Sun, Y. & Thiel, W. R. Ruthenium(II) phosphite complexes as catalysts for the hydrogenation of carbon dioxide. *ChemCatChem* **5,** 1340−1343 (2013).

[19] Himeda, Y., Onozawa-Komatsuzaki, N., Sugihara, H. & Kasuga, K. Simultaneous tuning of activity and water

solubility of complex catalysts by acid-base equilibrium of ligands for conversion of carbon dioxide. *Organometallics* **26,** 702−712 (2007).

[20] Wang, W. H., Hull, J. F., Muckerman, J. T., Fujita, E. & Himeda, Y. Second-coordination-sphere and electronic effects enhance iridium-(III)-catalyzed homogeneous hydrogenation of carbon dioxide in water near ambient temperature and pressure. *Energy Environ. Sci*. **5,** 7923−7926 (2012).

[21] Hull, J. F., Himeda, Y., Wang, W. H., Hashiguchi, B., Periana, R., Szalda, D. J., Muckerman, J. T. & Fujita, E. Reversible hydrogen storage using CO_2 and a proton-switchable iridium catalyst in aqueous media under mild temperatures and pressures. *Nat. Chem.* **4,** 383−388 (2012).

[22] Wang, W. H., Muckerman, J. T., Fujita, E. & Himeda, Y. Mechanistic insight through factors controlling effective hydrogenation of CO_2 catalyzed by bioinspired proton-responsive Iridium(III) complexes. *ACS Catal.* **3,** 856−860 (2013).

[23] Azua, A., Sanz, S. & Peris, E. Water-soluble Ir(III) N-heterocyclic carbene based catalysts for the reduction of CO_2 to formate by transfer hydrogenation and the deuteration of aryl amines in water. *Chem. Eur. J.* **17,** 3963−3967 (2011).

[24] Bertini, F., Gorgas, N., Stöger, B., Peruzzini, M., Veiros, L. F., Kirchner, K. & Gonsalvi, L. Efficient and mild carbon dioxide hydrogenation to formate catalyzed by Fe(II) hydrido carbonyl complexes bearing 2, 6-(diaminopyridyl) diphosphine pincer ligands. *ACS Catal.* **6,** 2889−2893 (2016).
